# Orthodontic Management of Congenitally Missing Maxillary Lateral Incisors: A Case Report

**DOI:** 10.1155/2014/731074

**Published:** 2014-03-09

**Authors:** Sergio Paduano, Iacopo Cioffi, Roberto Rongo, Antonello Cupo, Rosaria Bucci, Rosa Valletta

**Affiliations:** ^1^Department of the Health, University “Magna Graecia” Catanzaro, Viale Europa, Località Germaneto, 88100 Catanzaro, Italy; ^2^Department of Neuroscience, Section of Orthodontics, University of Naples Federico II, Via Pansini 5, 80131 Naples, Italy; ^3^Private Practice, Palomonte, 84020 Salerno, Italy

## Abstract

This case report describes the orthodontic treatment of a woman, aged 15 years, with permanent dentition, brachyfacial typology, with congenitally missing maxillary lateral incisors. Multibracket straightwire fixed appliance was used to open the space for dental implant placement, and treat the impaired occlusion. The missing lateral incisors were substituted with oral implants.

## 1. Introduction

The management of missing lateral incisor requires an integrated multidisciplinary approach [1]. Generally the choice between space opening with tooth replacement and space closure with canine substitution relies on several parameters to be considered before treatment planning.

Commonly the choice is related to occlusal relationship (i.e., overjet and overbite, molar relationship), facial typology and profile, arch length, and tooth size discrepancies. The morphology of the canine, in terms of size and shape, and its colour [2] also may address different treatment strategies. Finally, patient expectation and compliance can influence the treatment planning.

In case of unilateral tooth agenesis, space opening is often recommended to improve the aesthetics of patients and preserve smile symmetry. On the contrary, in case of bilateral agenesis, space closure and space opening could be both performed with respect to the issues previously reported [3–6].

Space opening is advised in low-angle subjects, whilst in high-angle individuals space closure should be preferred to preserve arch anchorage and avoid clock-wise rotation of the lower jaw. Retruded profiles should be better treated with space opening and tooth substitution, in order to improve labial sagittal relationships. This treatment strategy should be avoided in subjects with bimaxillary dental protrusion, in which it could result in worsening of the profile.

Molar relationship should be also considered. Molar class I or class III tendency should be better treated with space opening to preserve ideal occlusal anterior and posterior relationship (i.e., canine and molar relationship) and establish a solid angle class I. In case of full cusp or partial molar class II, space closure should be preferred to facilitate orthodontic biomechanics and reduce treatment duration. A stable molar class II and canine class I are then obtained. However, in case of arch length discrepancies extractions in the lower arch should be considered, thus obtaining a molar and canine class I.

Anterior relationship, that is, overjet and overbite, must be taken into account in terms of facilitation of biomechanics. Reduced overjet and increased overbite may easily be improved by space opening mechanics, whilst increased overjet and reduced overbite may benefit from space closure.

Shape and size of canines affect the possible rehabilitation choice. Differently from cases with large canines, in which space opening is advocated, small canines can be easily transformed in lateral incisors by using porcelain veneers or composite materials. The original position of the canine should be considered. Teeth closer to the midline are best candidate for incisor substitution.

The presence of third molars is an additional item supporting space closure mechanics. Finally, young individuals may preferably be treated with space closure to avoid frequent provisory prosthetic rehabilitation during adolescence.

In this case report we describe the orthodontic treatment of a girl, aged fifteen years, with permanent dentition, brachyfacial typology, with congenitally missing maxillary lateral incisors. Multibracket straightwire fixed appliance, along with cantilever mechanics, was used to open the spaces for oral implant placement and treat the impaired occlusion according to the principles previously examined. The missing lateral incisors were substituted with dental implants.

## 2. Case Presentation

### 2.1. Diagnosis and Treatment Plan 

**Figure 1:**
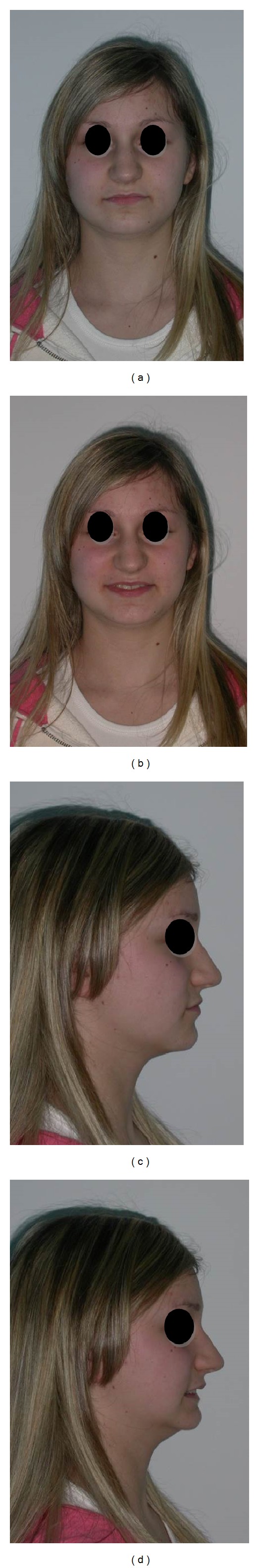
Extraoral photographs before treatment.

**Figure 2:**
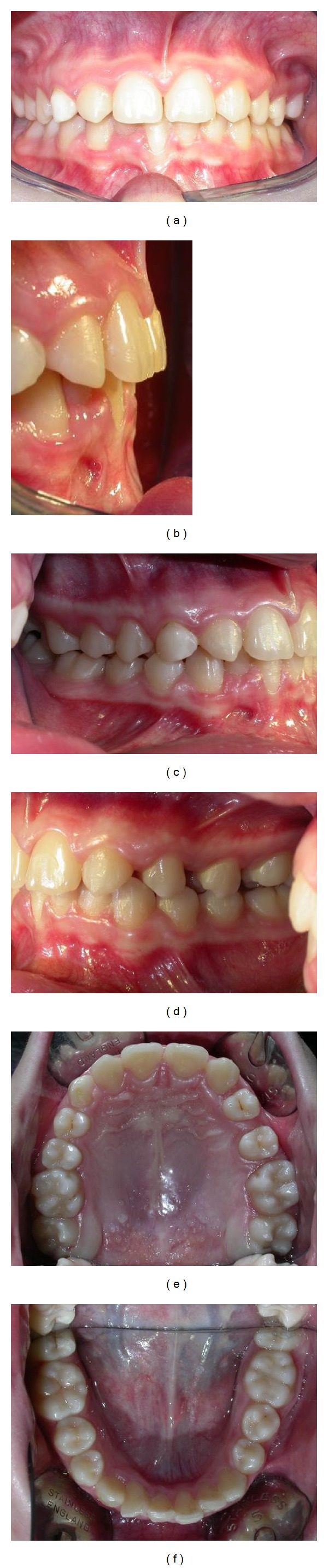
Intraoral photographs before treatment.

The extraoral and intraoral photographs of the patient are reported in Figures 1 and 2. The patient was 15 years old. She presented this objective problem list:

- missing maxillary lateral incisors;
- class I malocclusion;
- presence of the deciduous maxillary right canine;
- spacings in the left side;
- brachyfacial typology and retruded profile;
- slight arch length discrepancies;
- maxillary permanent canines close to midline;
- slight deviation of the upper midline.

She sustained a whiplash injury at the age of thirteen, due to a car accident, but she did not present signs or symptoms of temporomandibular disorders according to Research Diagnostic Criteria for Temporomandibular Disorders (RDC/TMD) [7, 8]. Oral parafuctions such as clenching were present and taken into account because of a possible relation with muscle pain [9].

**Figure 3:**
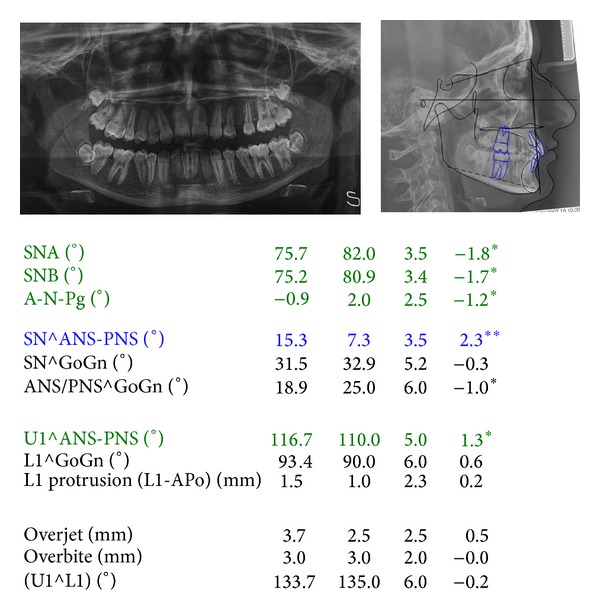
Cephalometric values and panoramic radiograph at the start of treatment. From left to right: value measured, average value from the population norm, standard deviation of the average value from the population norm, and difference from the extreme value of the population norm. Blue: values above the norm; green: values below the norm; black: values within the norm.

The cephalometric evaluation highlighted a brachifacial typology with a sagittal skeletal relationship of class I (Figure 3). The patient reported to have been treated at the age of 10 with a functional appliance (Sander Bite Jumping Appliance) to correct a skeletal class II malocclusion [10].

The treatment plan included the space opening of missing lateral incisors for implant placement and correcting her occlusion.

**Figure 4:**
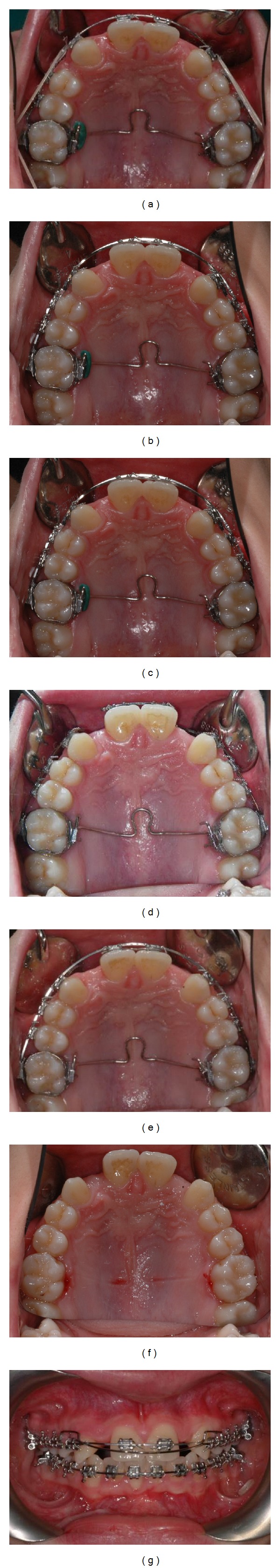
Progressive space opening for the maxillary lateral incisors ((a)–(f)). The distopalatal rotations of the canines were further corrected by using a .019′′ × .025′′ TMA sectional determining Burstone's 6th Geometry (g).

A fixed multibracket appliance was placed to align, level, and manage spacings of both upper and lower dental arches. Thermal Ni-Ti archwires were preferred to increase patient compliance and reduce initial discomfort [11]. The biomechanics and the progressive opening of the space for the maxillary lateral incisors are showed in Figure 4.

Initially, a .036′′ stainless steel transpalatal arch was modelled to correct molar rotations and to obtain additional anchorage. Later, compressed springs were applied to this appliance to obtain further proclination of the upper incisors [12].

Alignment of both dental arches was achieved by using multibracket appliance (Roth prescription, slot size .022′′ × .028′′ with heat activated Ni-Ti archwires (round .014′′ and round .016′′). Transbond XT (3 M Unitek Monrovia, US) adhesive primer was used for its strength [13] following the instructions of the manufacturer.

After aligning, the cuspids distalization was obtained by using interarches and intra-arch elastics on a round .018′′ AJ Wilcock Australian wire (regular +, G&H Orthodontics, Franklin, IN, US). The anchorage was preserved by placing the elastics between upper molars and canines during daytime and between lower molars and upper canines at nighttime. This pattern was easily accepted by the patients because during daytime the elastics were not visible. Superelastic coil springs for gaining space for maxillary lateral incisors were avoided in order to preserve the initial overjet.

The distalization of the canines resulted in a slight distopalatal rotation and a distal tip of the crowns. The distopalatal rotations were corrected by using a .019′′ × .025′′ TMA sectional determining Burstone's 6th Geometry [14], so that an ideal rotational effect was obtained without horizontal undesirable movements. To further obtain root uprighting and achieve an ideal placement of the roots for a proper site for implant rehabilitation, two cantilevers were used and applied to the central incisors segment. This allowed also for a better control of the overbite. The correction of midline discrepancy was mainly obtained by using a stainless steel .021′′ × .025′′ power arm positioned on the central incisors and shaped so that the force was applied close to the center of resistance of both teeth. This was finally attached with an elastomeric ligature to a TMA .019′′ × .025′′ cantilever inserted in the auxiliary buccal gingival tube of the band of the first left maxillary molar.

The anchorage for proper biomechanics was obtained by using full size stainless steel wires and a passive transpalatal arch.

**Figure 5:**
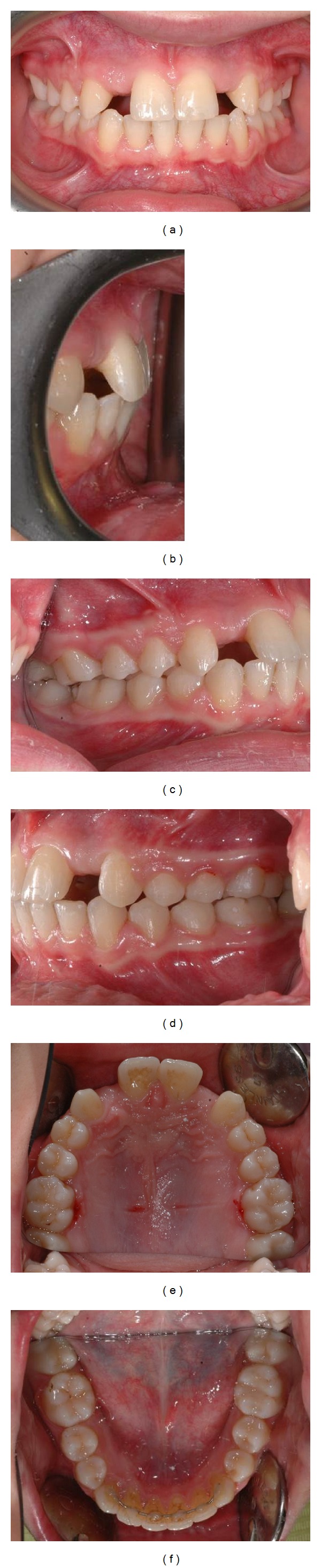
Intraoral photographs after treatment, before implant positioning.

**Figure 6:**
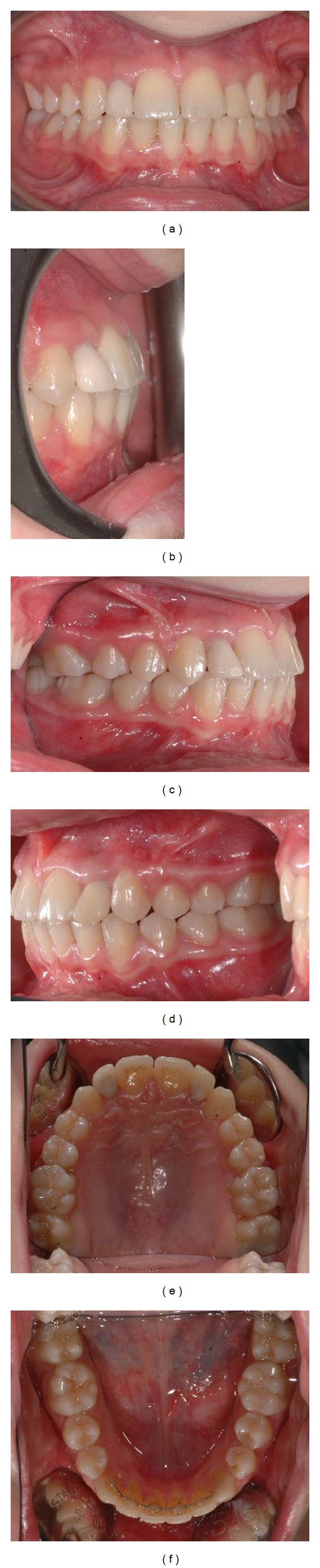
Intraoral photographs after prosthetic rehabilitation.

**Figure 7:**
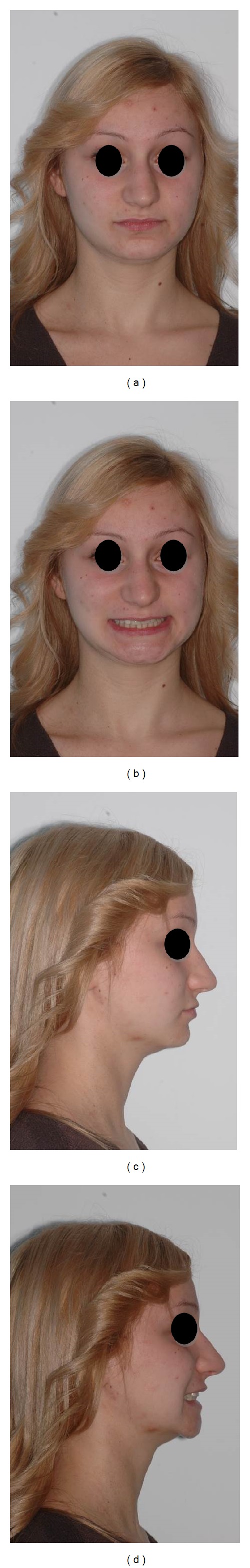
Extraoral photographs after treatment.

The treatment lasted approximately thirty months. Thereafter the patient was referred to the oral surgeon for the positing of the implants. The intraoral and extraoral photographs at the end of orthodontic treatment are presented in Figures 5–7.

Bone grafts from the extraction site of the lower left wisdom tooth were inserted by piezoelectric surgery in the maxillary lateral incisors sites. The grafts were fixed by using osteosynthesis screws in the implant sites and covered by absorbable membranes.

Intralock (Boca Raton, Fl, US) implant, 3.4 mm diameter, 11 mm length, was used. Provisory Maryland bridges were applied to preserve facial and smile aesthetics. After 6 months, gold abutments were fixed, with alumina-zirconia crowns (Figure 6).

## 3. Discussion

**Figure 8:**
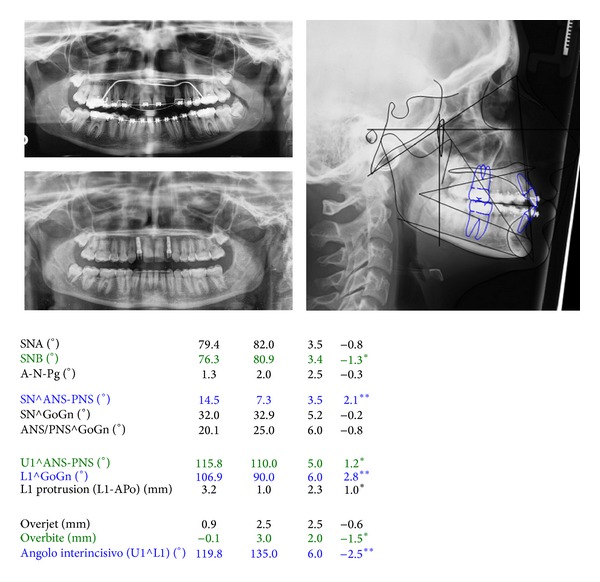
Cephalometric values just before the end of treatment and panoramic radiograph before final debonding and after implant placement. From left to right: value measured, average value from the population norm, standard deviation of the average value from the population norm, and difference from the extreme value of the population norm. Blue: values above the norm; green: values below the norm; black: values within the norm.

The major objectives of the treatment were achieved. Molar and canine class I relationship was achieved with overjet and overbite within the norms. The panoramic radiograph shows a good radicular parallelism and no signs of root resorption (Figures 6, 7, and 8). The clinical examination of the masticatory muscles and temporomandibular joints did not show any pathological signs or symptoms at completion of treatment.

The cantilever mechanics [15] allowed a correct repositioning of the roots of the maxillary incisors.

Conventional brackets were used because it has been suggested that self-ligating brackets are critical for obtaining an adequate torque control [16]. Also we used heat activated Ni-Ti archwires to reduce patient discomfort [11].

The results achieved were maintained during the retention period by means of a fixed lingual 33–43 retainer. The results achieved were substantially maintained at posttreatment control. Occlusal relationship and dental alignment were stable.
